# Endovascular brachytherapy combined with portal vein stenting and transarterial chemoembolization improves overall survival of hepatocellular carcinoma patients with main portal vein tumor thrombus

**DOI:** 10.18632/oncotarget.14520

**Published:** 2017-01-05

**Authors:** Tian-Zhu Yu, Wen Zhang, Qing-Xin Liu, Wen-Hui Li, Jing-Qin Ma, Zi-Han Zhang, Min-Jie Yang, Jian-Hua Wang, Bing Chen, Shao-Chong Zeng, Jian-Jun Luo, Ling-Xiao Liu, Zhi-Ping Yan

**Affiliations:** ^1^ Department of Interventional Radiology, Zhongshan Hospital, Fudan University, Shanghai, China; ^2^ Shanghai Institute of Medical Imaging, Shanghai, China; ^3^ Department of Radiotherapy, Zhongshan Hospital, Fudan University, Shanghai, China; ^4^ Department of Interventional Radiology, Yancheng Third People's Hospital, Southeast University, Yancheng, China

**Keywords:** hepatocellular carcinoma, main portal vein, tumor thrombus, endovascular brachytherapy, three-dimensional conformal radiotherapy

## Abstract

Hepatocellular carcinoma (HCC) patients with main portal vein tumor thrombus have a median survival time of only about 4 months. We therefore compared the safety and efficacy of endovascular brachytherapy (EVBT) and sequential three-dimensional conformal radiotherapy (3-DCRT). From a cohort of 176 patients, we treated 123 with EVBT using iodine-125 seed strands (group A) and the remaining 53 with sequential 3-DCRT (group B). Overall survival, progression free survival and stent patency characteristics were compared between the two groups. Our analysis demonstrated a median survival of 11.7 ± 1.2 months in group A versus 9.5 ± 1.8 months in group B (*p* = 0.002). The median progression free survival was 5.3 ± 0.7 months in groupA versus 4.4 ± 0.4 months in group B (*p* = 0.010). The median stent patency period was 10.3 ± 1.1 months in group A versus 8.7 ± 0.7 months in group B (*p* = 0.003). Therefore, as compared to sequential 3-DCRT, EVBT combined with portal vein stenting and TACE improved overall survival of HCC patients with main portal vein tumor thrombus.

## INTRODUCTION

Hepatocellular carcinoma (HCC) is the third leading cause of cancer mortality worldwide [1]. Portal venous invasion is frequently encountered with advanced stage HCC [2]. Main portal vein tumor thrombus (MPVTT) increases the metastasis risk, aggravates portal hypertension, and decreases the hepatopedal portal blood flow of the patients [3]. If untreated, the median survival time of these patients is only 2.7 months to 4 months [2].

The occurrence of MPVTT prevents hepatectomy and liver transplantation [4]. Sorafenib demonstrated survival benefits in recent phase III clinical trials and was considered for standard therapy in advanced HCC patients [5, 6]. However, high costs have limited its use in the developing countries [7]. External beam radiation therapy [8, 9] and trans-arterial radioembolization with yttrium-90 microspheres [9] treats HCC with portal vein thrombosis. However, the blood flow of obstructed MPV could not be restored promptly with either external or internal radiotherapy alone. Trans-arterial chemoembolization (TACE) with or without portal vein stenting can be performed safely in advanced HCC with MPV obstruction [10, 11]. Nonetheless, the efficacy of TACE for tumor thrombus, which often lacks tumor feeding arteries, is controversial [5, 6, 12]. Thus, novel effective treatment modalities need to be explored.

Recently, endovascular brachytherapy (EVBT) with Iodine-125 seed strand implantation was reported to be safe for advanced HCC with main portal vein tumor thrombosis [13–15]. Meanwhile, the combination of sequential three-dimensional radiotherapy (3-DCRT) with portal vein stenting and TACE was also reported to beneficial for these patients [16]. Since these two treatment regimens have not been compared, we analyzed the safety and efficacy of portal vein stenting and TACE combined with EVBT or sequential 3-DCRT to treat HCC with MPVTT in this study.

## RESULTS

### Patient data

Most patients included in this study were males with a mean age of 52.4 ± 9.9 years (range 28 – 75 years). Cirrhosis, secondary to hepatitis B was recorded for over 79% of the patients. We also recorded multifocal HCC in 136 (77.3%) patients and diffuse HCC in 40 (22.7%) patients. More than 60% patients demonstrated maximal diameter of HCC greater than 5 cm. Tumor thrombus that extended from the right intrahepatic portal vein branches into MPV was more frequently encountered than the left. Stenosis and occlusion of the MPV was found in 133 (75.6%) and 43 (24.4%) patients, respectively. The baseline characteristics of patients showed no significant difference between the two groups (Table 1).

**Table 1:** Baseline characteristics of patients

Characteristics	Group A (N = 123)	Group B (N = 53)	*p* value
Age (years) (SD)	52.6 (10.2)	51.7 (9.3)	0.278^c^
Sex (male/female)	113/10	46/7	0.296^d^
Etiology of cirrhosis (HBV/HCV/alcoholic/cholestasis)	98/14/9/2	35/11/6/1	0.267^d^
HCC morphology (multifocal/diffuse)	98/25	38/15	0.247^d^
HCC maximum diameter (cm) (≥5/<5)	79/44	34/19	0.992^d^
Location of TT(LIPV + MPV/RIPV + MPV)	42/81	15/38	0.447^d^
Degree of MPVTT^a^ (stenosis/occlusive)	93/30	40/13	0.984^d^
AFP (ng/ml) (>400/≤400)	80/43	33/20	0.724^d^
Child-Pugh grade (A/B)	111/12	45/8	0.306^d^
ECOG PS (0/1/2)	10/82/31	4/31/18	0.491^d^
Previous treatment (No/Resection/TACE/RFA/Combination therapy^b^)	81/12/16/6/8	30/4/10/7/2	0.237^d^

Abbreviations: AFP Alpha-fetoprotein, HBV hepatitis B virus, HCC hepatocellular carcinoma, HCV hepatitis C virus, LIPV left intrahepatic portal vein, MPV main portal vein, MPVTT main portal vein tumor thrombus, RFA radiofrequency ablation, RIPV right intrahepatic portal vein, SD standard deviation, TACE transarterial chembolization, TT tumor thrombus.

^a^ If the diameter of the filling defect exceeded 90% of MPV's on the transverse section image of contrast-enhanced CT or MRI before therapy, this patient's MPV was arbitrarily defined as occlusive.

^b^ Combination therapy means surgical resection followed by TACE or RFA or both and TACE combined with RFA

^c^ Independent *t* test was used

^d^ Chi-square test was used

### Portal vein stenting, EVBT, SPECT/CT scan and sequential 3-DCRT data

The mean length of the obstructed MPV was 55.4 ± 25.8 mm (range 10–170 mm) in Group A and 56.6 ± 23.6 mm (range 10–100 mm) in Group B (*P* = 0.747). After stent placement, the mean pressure of the MPV dropped from 40.6 ± 5.2 cm H_2_O (range 28–55 cm H_2_O) to 34.5 ± 5.0 cm H_2_O (range 25–44 cm H_2_O) (*P* < 0.001) in group A and from 41.8 ± 5.8 cm H_2_O (range 31–57 cm H_2_O) to 35.3 ± 4.9 cm H_2_O (range 26–45 cm H_2_O) (*P*< 0.001) in group B. A mean number of 16.1 ± 5.3 (range 6–26) Iodine-125 seeds were implanted in the MPV of group A patients. A mean 162.3 ± 21.8 Gy (range 81.6 –192.0 Gy) dose of radiation was prescribed to the tumor thrombus in group A based on the formula provided by the American Association Physicists in Medicine [17] and the Iodine-125 Radiation Field Distribution Calculation software (version 0.11, Shanghai Medical Radiation Research Institute) used by Zhang [18] and Chen [19]. The SPECT/CT scans showed that all stents and radioactive Iodine-125 seeds strands had been placed in the obstructed MPV correctly without displacement in the group A patients. Within 2-6 weeks, a mean radiation dose of 51.4 ± 8.4 Gy (range 20 – 66 Gy) was delivered by 3-DCRT to the group B patients.

### Tumor response to TACE procedures

A mean number of 3.3 ± 1.9 sessions of TACE (range 1–9) were performed in Group A and 3.6 ± 2.2 (range 1–10) in Group B (*P* = 0.231) patients. The mean dose of epirubicin and iodized oil used in the TACE procedure was 26.7 ± 7.1mg (range 10–40 mg), 9.5± 4.1ml (range 2–20 ml) in Group A and 26.0 ± 7.9mg (range 10–40 mg), 9.3 ± 4.3 (range 3–16 ml) in Group B (*P* = 0.557 and 0.771), respectively. The objective HCC response rate (CR + PR) was 19.5 % in group A and 17.0 % in group B (*P* = 0.693).

### Treatment-related complications

No complications related to stent deployment and Iodine-125 seeds strand implantation, such as intraperitoneal bleeding, stent displacement and radioactive seeds dislodgement, were recorded. Post-chemoembolization syndrome, including fever, vomiting and right upper abdominal pain, was observed in almost all patients. No statistical difference was found between the two groups. All the symptoms resolved within 3 – 5 days after symptomatic treatments. A transient increase of aminotransferase and bilirubin after the procedures was recorded. No grade 3 or 4 radiation-induced toxicity occurred.

### Overall survival analysis

During a mean follow-up time of 11.7 ± 8.3 months (range 1.2 – 32.0 months), 95 (77.2%) and 48 (90.6%) patients died in group A and B, respectively (*P* = 0.038). The mean and median survival times were 15.1 ± 1.0 months (95 % CI 13.2 – 17.1 months) and 11.7 ± 1.2 months (95 % CI 9.3-14.1 months) in group A compared to 10.4 ± 1.0 months (95 % CI 8.5 – 12.2 months) and 9.5 ± 1.8 months (95 % CI 5.9 – 13.1 months) in group B. The 12- and 24-month cumulative survival rates were 48.7% and 26.1% in group A and 31.4% and 3.4% in group B, respectively (*P* = 0.002) (Figure 1A). EVBT, stent patency and variceal bleeding were identified as independent predictors of patient's survival in both the univariate and multivariate analysis (Table 2).

**Figure 1A:**
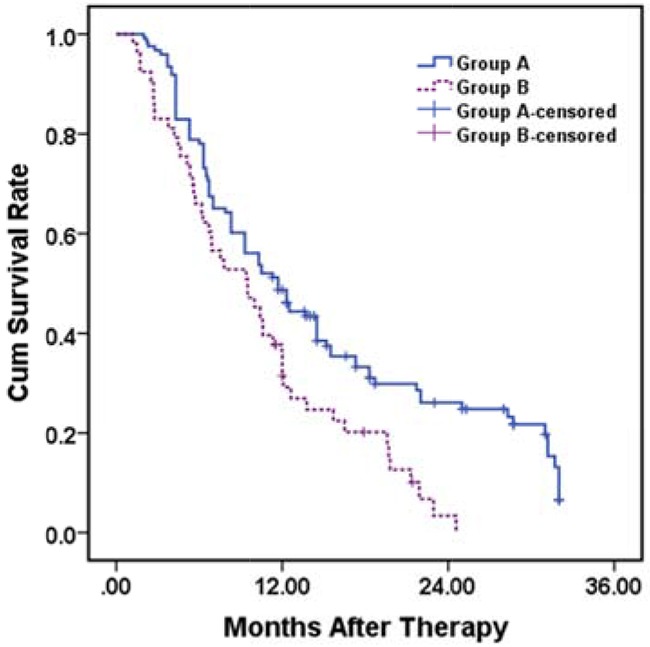
Kaplan–Meier analysis for overall survival in group A (with EVBT) versus group B (with 3-DCRT) Mean and median survival time were 15.1 ± 1.0 months (95 % CI 13.2 – 17.1 months) and 11.7 ± 1.2 months (95 % CI 9.3-14.1 months) in group A compared to 10.4 ± 1.0 months (95 % CI 8.5 – 12.2 months) and 9.5 ± 1.8 months (95 % CI 5.9 – 13.1 months) in group B, respectively. The 12- and 24-month cumulative survival rates were 48.7%, 26.1% in group A and 31.4%, 3.4% in group B, respectively (*p* = 0.002, log rank test).

**Table 2:** Predictors for survival in univariate and multivariate analysis

Variable	Univariate Analysis			Multivariate Analysis		
	HR	95%CI	p-Value	HR	95%CI	*p*-Value
Age (years) (≥55/<55)	1.080	0.775-1.506	0.649			
Sex (male/female)	1.042	0.576-1.884	0.893			
HCC morphology (multifocal/diffuse)	0.735	0.488-1.107	0.140			
Etiology of cirrhosis (With/Without hepatitis)	0.955	0.755-1.209	0703			
HCC maximum diameter (cm) (>=5/<5)	1.054	0.743-1.496	0.768			
Location of TT(LIPV + MPV/RIPV + MPV)	0.958	0.673-1.363	0.811			
Degree of MPVTT (stenosis/occlusive)^a^	0.876	0.597-1.286	0.500			
AFP (ng/ml) (>400/≤400)	1.328	0.934-1.889	0.114			
Child-Pugh grade (A/B)	0.824	0.495-1.371	0.455			
ECOG PS (0 and 1/2)	1.098	0.827-1.457	0.518			
Previous treatment (Yes/No)	1.080	0.922-1.264	0.341			
Therapy (EVBT/3D-CRT)	0.581	0.407-0.830	0.003*	0.649	0.448-0.938	0.022*
Stent (Patent /Occlusive)	0.477	0.316-0.718	<0.001*	0.553	0.360-0.849	0.007*
HCC response (CR + PR/SD + PD)	0.994	0.649-1.522	0.979			
Variceal bleeding after therapy (Yes/No)	1.930	1.338-2.784	<0.001*	1.907	1.316-2.763	0.001*
Liver function decompensation after therapy (Yes/No)	1.104	0.721-1.690	0.648			

Abbreviations: AFP Alpha-fetoprotein, CI confidence interval, CR complete response, ECOG PS Eastern Cooperative Oncology Group Performance Status, EVBT endovascular brachytherapy, LIPV left intrahepatic portal vein, HCC hepatocellular carcinoma, HR hazard ratio, MPV main portal vein, MPVTT main portal vein tumor thrombus, PD progressive disease, PR partial response, RIPV right intrahepatic portal vein, SD stable disease, TT tumor thrombus

* A *p* value <0.05 indicated a significant difference

^a^ Diameter of the filling defect exceeded 90 % of MPVs on the transverse section image of contrast-enhanced CT or MRI before therapy was defined as occlusive

### Progression free survival analysis

During the course of the study, occurrence of either intra-hepatic/extra-hepatic HCC spread, variceal bleeding, liver function decompensation or occurrence of more than one of these events were observed in 59 (48.0 %), 30(24.4%), 11 (8.9%) and 12 (9.5%) in group A and 21(39.6 %), 16 (30.2 %), 7 (13.2 %) and 4 (7.5%) patients in group B, respectively (*P* = 0.752). The mean and median progression free survival time were 5.8 ± 0.3 months (95% CI 5.3 – 6.4 months) and 5.3 ± 0.7 months (95% CI 3.8–6.6 months) in group A compared to 4.7 ± 0.4 months (95 % CI 4.0 – 5.4 months) and 4.4 ± 0.4 months (95 % CI 3.6 – 5.2 months) in group B (*P* = 0.010) (Figure 1B).

**Figure 1B:**
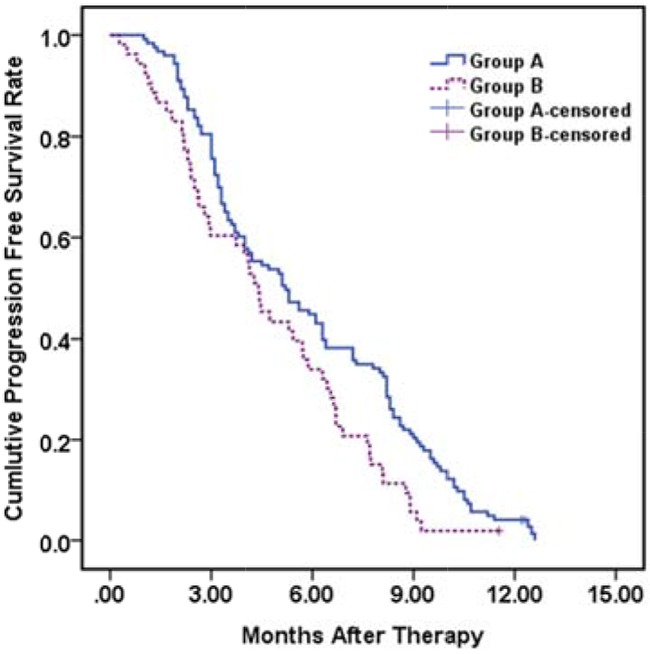
Kaplan–Meier analysis for progression free survival in group A (with EVBT) versus group B (with 3-DCRT) Mean and median progression free survival times were 5.8 ± 0.3 months (95% CI 5.3 – 6.4 months) and 5.3 ± 0.7 months (95% CI 3.8–6.6 months) in group A compared to 4.7 ± 0.4 months (95 % CI 4.0 – 5.4 months) and 4.4 ± 0.4 months (95 % CI 3.6 – 5.2 months) in group B (*p* =0.010, log rank test).

### Stent patency data

During the follow-up, stent occlusion was observed in 87 (70.7%) patients in group A and 41 (77.4%) patients in group B (*P* = 0.365). The mean and median stent patency periods were 14.7 ± 1.0 months (95 % CI 12.7–16.8 months) and 10.3 ± 1.1 months (95 % CI 8.1–12.5 months) in group A and 9.6 ± 0.8 months (95 % CI 8.1–11.2 months) and 8.7 ± 0.7 months (95 % CI 7.4 –10.0 months) in group B, respectively. The 12- and 24-month cumulative stent patency rates were 46.5% and 25.7 % in group A and 29.8% and 0% in group B, respectively (*P* = 0.003) (Figure 1C).

**Figure 1C:**
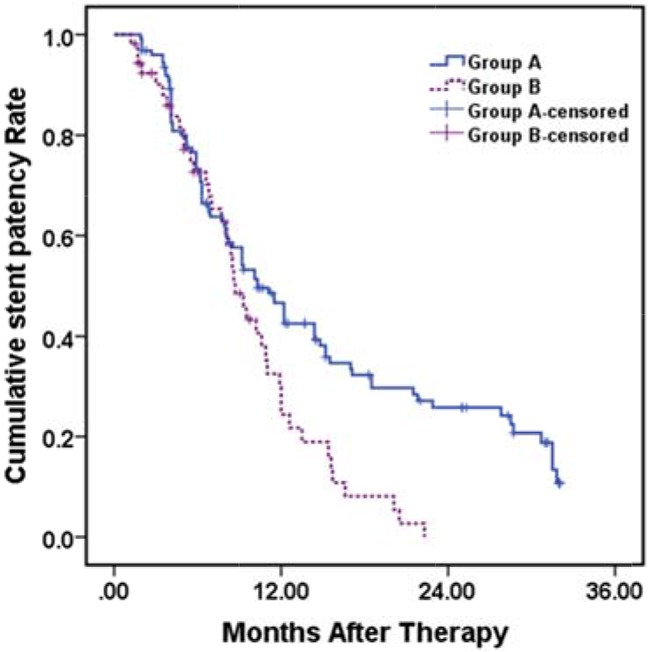
Kaplan–Meier analysis for stent patency period in group A (with EVBT) versus group B (with 3-DCRT) Mean and median stent patency period were 14.7 ± 1.0 months (95 % CI 12.7–16.8 months), 10.3 ± 1.1 months (95 % CI 8.1–12.5 months) in group A and 9.6 ± 0.8 months (95 %CI 8.1–11.2 months), 8.7 ± 0.7 months (95 % CI 7.4 –10.0 months) in group B, respectively. The 12- and 24-month cumulative stent patency rates were 46.5%, 25.7 % in group A and 29.8%, 0% in group B, respectively (*p* = 0.003, log rank test).

## DISCUSSION

Although the survival of patients with HCC has improved during recent decades [1], the prognosis of HCC that is complicated by MPVTT remains extremely poor [4]. The optimal treatment modality for this group of HCC patients has not yet been established [2].

Sorafenib was recommended for advanced HCC [5, 6] and TACE was reported to benefit patients with HCC and portal vein tumor thrombosis [10]. However, the patients treated with TACE alone [20], sorafenib monotherapy [21] and TACE combined with sorafenib [22–24] demonstrated poor overall survival times of 4.1, 3.0 and 3.0–7.0 months, respectively. To further the survival benefit for these patients, restoration of the hepato-petal portal vein blood flow and reduction of tumor thrombus burden were postulated. Portal vein stenting was used widely to treat MPV obstruction caused by both benign and malignant diseases [11, 16]. However, in-stent tumor growth and neointimal hyperplasia caused stent failure. Radiation therapy inhibited tumor cell proliferation and induce apoptosis [13, 25]. Especially, continuous low dose rate radiotherapy had anti-neointimal hyperplasia effect that could prolong the patency period of the stent [26]. Moreover, low dose rate of radiation had been reported to decrease the incidence of metastasis by altering the immunophenotype of the tumor cells [27, 28]. Most importantly, EVBT and sequential 3-DCRT were capable of inhibiting MPVTT [14–16, 29]. However, MPVTT treated by these two different types of radiation had never been directly compared.

In our cohort, Iodine-125 seeds strands were successfully placed in the obstructed MPV of group A patients. The maximal tolerance radiation dose of the vessels was reported to be more than 800 Gy [30]. In our study, a mean of 162.3 ± 21.8 Gy radiation dose was tolerated by all group A patients. Although this dose was higher than that in group B patients, no grade 3 or 4 radiation-induced toxicity was observed during the follow-up. This was probably because the radiation emitted by Iodine-125 seed was at a low dose rate and short tissue-penetrating distance.

Compared with external radiotherapy, brachytherapy with Iodine-125 seed implantation had the following advantages: (i) It had highly accumulated radiation within the tumor area without serious damage to the surrounding normal tissue; (ii) Sustained radiation inhibited tumor cell proliferation and induced apoptosis; (iii) It was not affected by the patient's respiration motion; (iv) A low dose rate radiation decreased the incidence of metastasis by altering the immunophenotype of tumor cells [28] and (v) It was convenient to patients as it was a one-stop treatment method.

Our data showed that the overall survival extended significantly in group A patients. Multivariate analysis showed that patients that were treated with EVBT with patent stents and no variceal bleeding had better survival (Table 2). The longer survival time in group A patients can be attributed to the anti-tumor and anti-neointimal hyperplasia effects of sustained low dose rate radiation emitted by the Iodine-125 seeds strands [26]. These prolonged the patency period of the stent and decreased the risk of second variceal bleeding.

Direct MPV puncturing for radioactive seed implantation was reported to treat MVPTT [31]. Iodine-125 seeds were aligned linearly and sealed into a 4-F catheter continuously to construct a seeds strand in our study to prevent seed dislodgment and ensure that the target lesion was covered by the radiation. Furthermore, single portal vein puncture site reduced bleeding complications. In fact, there were no bleeding complications recorded in Group A. We observed that the hepato-pedal portal flow was restored and the portal vein pressure decreased immediately after stenting. This increased the safety of subsequent TACE in patients with compromised portal blood supply [12].

Since this was a single-center retrospective study, potential selection bias cannot be excluded. Therefore, a well-designed prospective randomized trial with sufficient sample size is necessary to confirm the efficacy and superiority of portal vein stenting and TACE combined with EVBT for the treatment of patients with HCC and MPV tumor thrombus. Recently, Matsuo Y and colleagues reported that SBRT with hypofractionated technique might be superior to conventional three-dimensional conformal radiotherapy for the treatment of PVTT [35]. However, the SBRT technique was not available for our research study and hence in future SBRT and brachytherapy need to be comparatively investigated.

In conclusion, this study showed that portal vein stenting and TACE combined with EVBT could be performed safely in patients with HCC and MPVTT. Compared to sequential 3-DCRT, Iodine-125 seeds strands implantation significantly prolonged stent patency, extended the progression free survival time and improved the overall survival of patients with HCC and MPVTT.

## MATERIALS AND METHODS

### Study design and patient selection

**Figure 2:**
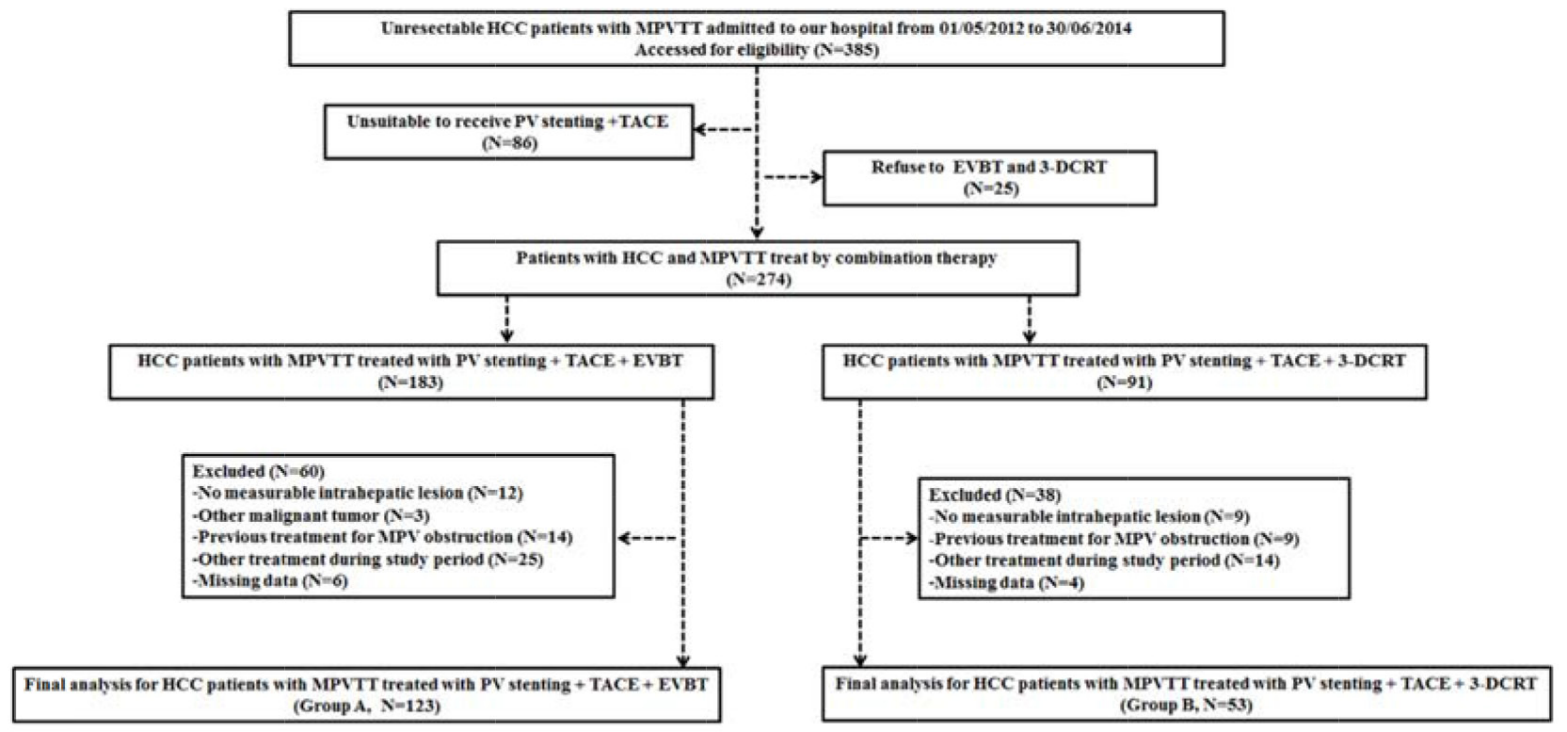
The flow diagram of patients enrolled in the study EVBT = Endovascular brachytherapy, 3-DCRT = Three-dimensional conformal radiotherapy, HCC = Hepatocellular carcinoma, MPV = Main portal vein, MPVTT = Main portal vein tumor thrombus, PV = Portal vein, SMV = Superior mesenteric vein, SV = Splenic vein, TACE = Transarterial chemoembolization, TT = Tumor thrombus.

**Figure 3:**
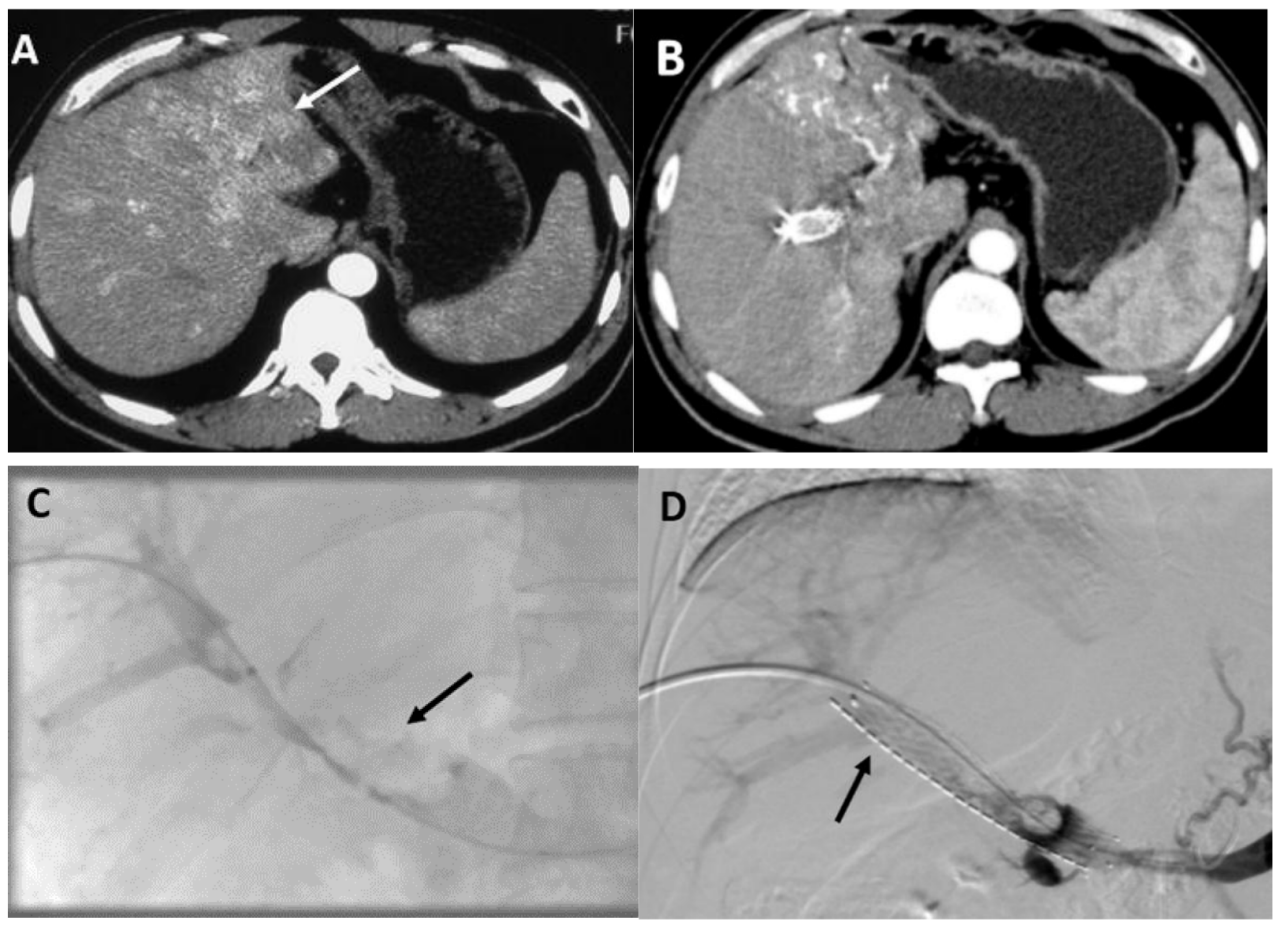
Images of portal vein stenting and TACE combined with endovascular brachytherapy performed in a 39-year-old male patient (group A) **A**. An invasive HCC (white arrow) detected on the left lobe by the enhanced abdominal CT scan before therapy. **B**. Image of an enhanced abdominal CT scan performed one month after the first therapy. Atrophic left lobe of liver and partial response of HCC to TACE was observed. **C**. The tumor thrombus (black arrow) in his MPV was observed on the direct portography after his right patent intrahepatic portal vein branch was punctured. **D**. Image captured after a 14/100 mm stent and iodine-125 seed strand that was loaded with 20 radioactive seeds (black arrow) had been implanted in his MPV showing restoration of the flow of the obstructed MPV.

This study was approved by the ethics committee and institutional review board (2009-080) of our institution. All procedures were followed in accordance with the ethical standards of the responsible committee on human experimentation (Zhongshan Hospital, Fudan University, China) and the Helsinki Declaration of 1975, as revised in 2008 (5). Informed consent was obtained from all patients included in the study. 385 patients diagnosed with HCC and MPVTT between May 2012 and June 2014 were referred to our institution. Among them, 86 patients were judged unsuitable to receive PV stenting and TACE (patients with Child-Pugh classification C or with Eastern Cooperative Oncology Group performance status of 3 or 4) and 25 patients refused EVBT or 3-DCRT. Therefore, 183 patients underwent EVBT combined with portal vein stenting and TACE and 91 patients received sequential 3-DCRT combined with portal vein stenting and TACE. Patients with no measurable intrahepatic lesions (12 in EVBT group and 9 in 3-DCRT group), other concurrent malignancies (3 in EVBT group), previous MPVTT treatment history (14 in EVBT group and 9 in 3-DCRT group), other treatments after the procedure (25 in EVBT group and 14 in 3-DCRT group) and missing follow-up clinical data (6 in EVBT group and 4 in 3-DCRT group) were excluded. Ultimately, 123 patients treated with EVBT (Group A) and 53 with 3-DCRT (Group B) were included in the final data analysis (Figure 2). Other eligible criteria were as follows: (i) age >18 years; (ii) HCC was diagnosed according to the European Association for the Study of Liver/American Association for the study of Liver Disease guidelines [32]; (iii) tumor thrombus, a low-attenuation intraluminal filling defect extending from intrahepatic portal vein branches adjacent to the primary tumor into MPV was confirmed by contrast-enhanced abdominal computer tomography (CT) or magnetic resonance imaging (MRI) (Figure 3A); (iv) patency of one or two of the second-order intrahepatic portal vein branch (v) Child-Pugh classification grade A or B; (vi) Eastern Cooperative Oncology Group (ECOG) performance status less than or equal to 2 and (vii) No contraindication for TACE, such as HCC burden >70 % of total liver volume, high-flow intrahepatic arterial venous shunt and/or serious coagulant disorder. Patients were excluded from this study if (i) no measurable intrahepatic lesion could be observed; (ii) they had concurrent malignancy other than HCC; (iii) previous therapy was performed for MPV obstruction (such as stent placement, radiotherapy or sorafenib); (iv) they received other treatment (radiofrequency ablation or molecular targeted drug therapy) besides the above mentioned therapy during the course of this study or (v) the data of follow-up were missing. Either EVBT or radiation was considered to be beneficial for patients with HCC and tumor thrombus in the main portal vein but not as a standard therapeutic option. According to the Chinese expert consensus on multidisciplinary diagnosis and treatment of hepatocellular carcinoma with portal vein tumor thrombus, the treatment choices for patients with MPVTT include surgery, radiotherapy and/or TACE depending on the patient's preference [33]. Before the procedure, the benefits and potential adverse events related to endovascular brachytherapy (EVBT) and radiation were explained in detail to the patients and informed consents were signed. Therefore, the decision to receive EVBT or radiation was entirely based on the patient's own will.

### Stent and Iodine-125 seed characteristics

**Figure 4:**
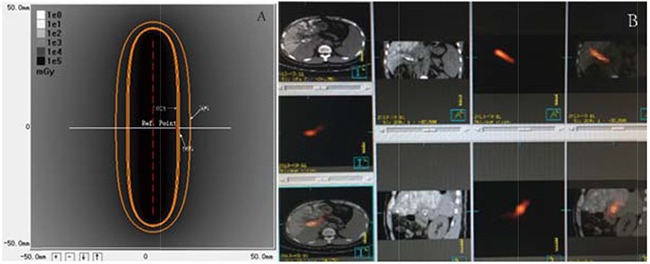
Images of dosimetry of a Iodine-125 seeds strand and SPECT/CT **A**. Dosimetry of a seed strand containing 16 Iodine-125 seeds. The isodose contours are: 100% (62.5Gy, reference point, red dot), 90% (56.2Gy), and 50% (31.2Gy). The 240 day accumulation dose was141.6Gy. **B**. Image of a SPECT/CT scan performed 1 day after the procedure. Stent and I-125 seed strands were implanted correctly in the MPV without displacement. Radiation emitted by a I-125 seed strand was distributed homogeneously and completely covered the target lesion.

Self expanding Nitinol stents (Luminxx III, Bard, Covington, GA) with a diameter of 12–14-mm and length of 60–100mm were used in this study. The model 6711 Iodine-125 seeds (XinKe, Shanghai, China) used in this study were a cylindrical brachytherapy source with an active length of 3.25 mm encapsulated by titanium. The diameter and the length of the titanium capsule were 0.8 mm and 4.5 ± 0.5 mm, respectively. The radioactivity of each Iodine-125 seed was 25.9 MBq with a half-life of 59.4 days. The principal photon emissions were 27.4-, 31.4-keV X-ray and 35.5-keV gamma-ray. The half-value thickness of tissue for Iodine-125 seeds was 17 mm and the incipient dose rate was 7 cGy/h. Seeds were arranged linearly and sealed into a 4-F catheter continuously to construct an Iodine-125 seeds strand. The dose reference point was chosen at 10mm from the axis of the iodine-125 seed strand source (Z=0, r=10 mm). The 240 days accumulating dose of the reference point was calculated by an iodine-125 Radiation Field Distribution Calculation software (version 0.1, Institute of Radiation Medicine, Fudan University, Shanghai, China) based on the American Association of Physicists in Medicine TG43U1 brachytherapy formula (Figure 4A).

### Portal vein stenting and Iodine-125 seeds strand placement procedures

After local anesthesia, a tumor-free second-order branch of the intrahepatic portal vein was punctured using a 22-gauge Chiba needle (Cook, Inc, Bloomington, Indiana) guided by ultrasound. A 6-F NEFF set (Cook) was introduced into the intrahepatic portal vein over a 0.018-inch wire (Cook). Then, a 0.035-inch, 150-cm-long wire (Terumo, Tokyo, Japan) combined with a 4-F Cobra catheter (Cordis, Miami Lakes, FL) was manipulated to cross the stenotic segment to the superior mesenteric vein (SMV) through the outer cannula of the NEFF set. The Cobra catheter was removed and the outer cannula of the NEFF set was replaced by a 7-F, 23-cm-long sheath (Cordis) over the wire. Portography was performed to measure the diameter and length of the stenotic segment by a 5-F calibrated pigtail catheter (Cook) placed in the splenic vein (Figure 3C). The portal vein pressure was measured at the distal and the proximal end of the obstructed MPV. If gastroesophageal varices were opacified, the left gastric vein or/and short gastric vein were embolized with coils (Cook). The number of Iodine-125 seeds that needed to be implanted was calculated by the following formula: N = length of obstructed MPV (mm)/4.5 + 4. After intravenous administration of 50 U/kg heparin (XingYi, Shanghai, China), two 0.035-inch, 260-cm-long stiff wires (Terumo) were inserted into SMV through the 7-F sheath. After the sheath had been removed, the outer cannula of the NEFF set and a self-expandable stent was introduced to the MPV over one of the stiff wires. The stent was deployed from distal MPV into the patent intrahepatic portal vein. Through the outer cannula of the NEFF set, the Iodine-125 seeds strand was delivered to the target position and released between the stent and the MPV. Portography and pressure measurements were performed again (Figure 3D). Finally, the transhepatic puncture track was occluded by coils (Cook).

### TACE methodology

To identify all the feeding arteries of the tumor, angiography of the celiac, hepatic, superior mesenteric, left gastric and bilateral inferior phrenic artery was performed by a 5-F RH catheter (Cook). The target artery was catheterized with a 2.7-F microcatheter (Renegade, Boston Scientific, Natick, MA). 10–50 mg/m^2^ of epirubicin (Pharmorubicin, Pfizer, New York, NY) was mixed with 5–20 ml of iodized oil (Lipiodol Ultrafluide, Laboratoire Guerbet, Aul-nay-sous-Bois, France) by using a pumping method. The dose of epirubicin and iodized oil was determined based on the patient's liver function and tumor vascularity. Under fluoroscopic monitoring, the mixture was infused at a rate of 0.5–1 ml/min through the microcatheter until stasis flow in the tumor vascularity was achieved. Finally, gelatin sponge (Jingling, Jiangsu, China) was used to embolize the feeding artery.

### Post-procedure management strategy

After the procedure, tropisetron hydrocloride (Novartis Pharma Schweiz AG, 5 mg/day), omeprazole (Changzhou Pharmacectic, Jiangsu, China 40 mg/day) and ornithine aspartate (Merz Pharma, GmbH & Co. KGaA, Frankfurt, Germany, 10 g/day) were administered intravenously to all patients for 3–5 days. Pain and fever attributed to post-embolization syndrome were controlled individually using non-steroidal anti-inflammatory drugs or opioids. 5,000 U of low molecular-weight heparin (XinYi, Shanghai, China) was injected subcutaneously twice a day. One day after the procedure, single-photon emission computer tomography (SPECT) combined with a CT (SPECT/CT) scan was performed to evaluate the distribution of radiation emitted by the Iodine-125 seeds strand implanted in the MPV (Figure 4B). Warfarin (XinYi, Shanghai, China), starting with 2.5 mg every day, was prescribed to all the patients, 3 days after the procedure for 6 months and the dosage was adjusted based on the coagulation function test (international normalized ratio = 1.8–2.0) performed weekly.

### Sequential 3-DCRT methodology

The method of stent implantation combined with TACE and the protocol of post–procedure management in group B was the same as in group A. Sequential 3–DCRT was performed two weeks after the above mentioned therapy. The CT data of the patients was transferred to a 3-DCRT treatment planning system (Pinnacle 7.6C). The gross target volume (GTV) included the stent. The CTV was defined as the volume of stent placed in the MPV (GTV) plus a small margin of 3–5 mm. Intra-hepatic tumor was not included in the CTV. The planning target volume (PTV) was CTV plus 5–10 mm margin to account for daily setup error and target motion. A single PTV was used throughout the treatment course without any reduction. The total dose was planned in order to achieve the 90% isodose curve covering 100% PTV. A daily dose of 2.0 Gy with 5 fractions per week was administered until the total prescribed dose had been delivered. Patients were assessed for toxicities on a weekly basis during 3-DCRT.

### Follow-up analysis

All patients were followed up at a 4-6-week interval until death or their last follow-up (before June 30, 2015). The response of HCC and stent patency was evaluated by abdominal contrast enhanced CT scan (Figure 3B). According to the modified response evaluation criteria in solid tumor (mRECIST) as recommended by American Association for the Study of Liver Diseases [34], the response was classified as complete response (CR), partial response (PR), stable disease (SD) and/or progressive disease (PD). Objective response was defined as the sum of CR and PR. Laboratory tests were performed to evaluate liver and renal function, blood cell count, and coagulation parameters.

Repeated TACE was performed either on detecting enhancement of the residual tumor on the arterial phase, occurrence of a new lesion or both. Indirect portography was undertaken to evaluate the patency of the stent during repeated TACE. TACE was suspended if there was decompensation of liver function or decline in clinical status.

### Efficacy and safety assessment

A low-attenuation intraluminal filling defect that extended from the intrahepatic portal vein branches adjacent to the primary tumor into the MPV was defined as portal vein tumor thrombosis (PVTT).

Overall survival, progression free survival, stent patency period and treatment-related adverse events were compared between the two groups. Survival time was defined as the period from the day of stent placement to patients’ death or to their last follow-up. Occurrence of intrahepatic/extrahepatic HCC spread, variceal bleeding and liver function decompensation (e.g., uncontrolled ascites or hepatic encephalopathy) were considered as disease progression. Therefore, progression free survival was the time from first therapy to the presence of one or more of above mentioned events or to the patient's death. Stent occlusion was defined as no contrast medium visualized inside the stent on the portal phase of the contrast enhanced CT scan or indirect portography during the repeat TACE procedures. The stent patency period was calculated as the interval of the day of stent placement and stent occlusion or the day of last follow-up. Treatment-related adverse events were scored according to the Common Terminology Criteria for Adverse Events, version 3.0 [35].

### Statistical analysis

Continuous variables were presented as mean values ± standard deviation and compared by the independent or paired sample *t* test. Categorical variables were presented as frequencies and compared using the chi-square test. The overall survival, progression free survival and stent patency period were analyzed with the Kaplan–Meier curves and log-rank test. A *P* value <0.05 was considered statistically significant. Variables that achieved statistical significance (*P* < 0.05) in univariate analysis were subsequently assessed by multivariate analysis by Cox proportional hazards model. A stepwise regression procedure was used to determine the factors that were major independent predictors for survival. SPSS version 19.0 software (SPSS, Chicago, IL) was used for statistical analysis.
